# Working Memory Training in Schizophrenia and Healthy Populations

**DOI:** 10.3390/bs4030301

**Published:** 2014-09-03

**Authors:** Linette Lawlor-Savage, Vina M. Goghari

**Affiliations:** Clinical Neuroscience of Schizophrenia Laboratory, Departments of Psychology and Psychiatry, Hotchkiss Brain Institute, University of Calgary, Clinical Psychology, 2500 University Drive NW, Calgary, AB T2N 1N4, Canada; E-Mail: savagel@ucalgary.ca

**Keywords:** schizophrenia, working memory, cognitive remediation, dual n-back, intelligence

## Abstract

Cognitive deficits are consistently demonstrated in individuals with schizophrenia. Cognitive training involves structured exercises prescribed and undertaken with the intention of enhancing cognitive abilities such as attention, memory, and problem solving. Thus, cognitive training represents a potentially promising intervention for enhancing cognitive abilities in schizophrenia. However, cognitive training programs are numerous and heterogeneous, hence, the generalizability of training related outcomes can be challenging to assess. This article will provide a brief overview of current literature on cognitive training and explore how knowledge of working memory training in healthy populations can potentially be applied to enhance cognitive functioning of individuals with schizophrenia.

## 1. Introduction

Individuals with schizophrenia commonly demonstrate a compromised neurocognitive profile [1] and have been found to score one to two standard deviations below healthy individuals, or other individuals with a psychiatric disorder, on measures of cognitive performance [2]. Prior to diagnosis or symptom onset, schizophrenia patients score, on average, half a standard deviation lower than healthy populations on standardized IQ tests [3]. In a recent longitudinal analysis of IQ scores from childhood (ages 7, 9, 11, and 13) through to adulthood (age 38), individuals diagnosed with schizophrenia scored, on average, 9-points lower than those not later diagnosed with schizophrenia [4]. Furthermore, by adulthood, the schizophrenia group demonstrated a 6-point drop in IQ compared to childhood scores, whereas IQ did not change over time in the healthy group [4]. Findings were not accounted for by antipsychotic medication [4]. Facets of cognition identified as impaired in schizophrenia include attention, processing speed, working memory, verbal and visual learning and memory, reasoning and problem solving, verbal comprehension, and social cognition [5]. Cognitive impairment is considered a core feature of schizophrenia rather than a consequence of symptoms or pharmacological treatments [6], and likely represents a genetic marker of schizophrenia [7]. Furthermore, impaired cognitive ability is currently the strongest correlate of poor day-to-day functioning for individuals with schizophrenia [8]. Deficits in attention, working memory, and executive functioning have been associated with poor work skills, interpersonal communications, and community functioning [9], and unemployment [10]. Additionally, cognitive impairment and negative symptoms are, while distinct, highly correlated [11] such that a deficit in cognitive ability may contribute to more severe negative symptoms.

Robust deficits specifically in working memory have been identified in schizophrenia patients relative to healthy controls [12,13]. Working memory refers to the temporary storage and manipulation of perceived information, and is crucial for learning, reasoning, and comprehension of language [14]. As described in two comprehensive meta-analyses, clear deficits in working memory are present in schizophrenia across a number of measures, specifically, tasks assessing maintenance and/or manipulation of auditory, visual, lexical, or semantic information [12,13]. Specifically, difficulties with encoding and maintenance appear in schizophrenia across a range of visual-spatial and auditory tasks [13]. Working memory difficulties are not accounted for by symptoms such as disorganization or distractibility during acute psychotic phases of the disorder [12]. Compromised working memory has negative consequences for overall functioning in schizophrenia. For example, working memory deficits predict poor community functioning, decreased self-care and health maintenance activities, compromised vocational and social functioning, poor overall quality of life, and overall disability in schizophrenia [15]. Hence, improving working memory in schizophrenia could have wide-spread implications on day-to-day functioning for patients.

The aim of the present review is to explore remediation options for cognitive deficits in schizophrenia, focusing on working memory training as a method to enhance working memory. We provide an overview of working memory training programs, with an emphasis on dual n-back working memory training, a program with demonstrated benefit to working memory in healthy populations. We then outline working memory training studies in schizophrenia, and provide recommendations for investigation of dual n-back working memory training in schizophrenia.

## 2. Cognitive Remediation in Schizophrenia

Given that attention, working memory, and executive functioning are key cognitive processes associated with functional impairment in schizophrenia, [8,9,10] these areas represent reasonable targets of cognitive remediation. A formal definition of cognitive remediation for schizophrenia emerged from the 2010 Cognitive Remediation Experts Workshop as, “a behavioral training based intervention that aims to improve cognitive processes (attention, memory, executive function, social cognition or metacognition) with the goal of durability and generalization” [16]. With this definition in mind, we review the efficacy and limitations of current programs aimed to remediate cognitive abilities in individuals with schizophrenia.

Numerous reviews and meta-analyses describe benefits of cognitive remediation in schizophrenia [16,17,18,19,20,21,22,23,24,25,26,27]. Broadly, findings indicate that cognitive remediation is consistently associated with moderately improved cognitive performance [16,17,18,19,20,21,22,23,24,25,26,27]. Specifically, a meta-analysis of 40 studies totaling over 2100 participants yielded a Cohen’s *d* effect of cognitive remediation on global cognition of 0.45 (for summary table see [16]). When particular cognitive domains were considered separately, effects on attention, speed of processing, and visual learning and memory were small (*d* = 0.25, *d* = 0.26, and *d* = 0.15, respectively, with only visual learning and memory not statistically significant), with moderate effects on verbal learning and memory (*d* = 0.41), verbal working memory (*d* = 0.35) and reasoning/problem solving (*d* = 0.57). Therefore, within a broad range of cognitive processes, memory and reasoning/problem solving are most impacted by cognitive training.

However, the heterogeneous nature of cognitive remediation programs represents a key weakness in investigating and implementing empirically validated cognitive remediation. For example, Wykes and colleagues’ meta-analysis [16] represented 14 different forms of cognitive remediation ranging from self-talk strategies to computerized drill-and-practice techniques. Drill and practice approaches require the participant to repeatedly practice a specific task with improved performance expected with increased practice. Tasks are typically computerized and adapt to performance such that difficulty increases when a threshold of ability is reached, or decreases if the participant struggles with the task. The goal of drill and practice training is to improve performance on a particular core ability, such as attention or working memory [28]. Such a training technique could help the participant improve performance on a vigilance task which, in theory, could transfer to improvement on real-world tasks requiring sustained attention (e.g., attending to a co-worker’s speech; maintaining focus despite distraction). Conversely, strategy-based techniques aim to improve ability by introducing tools that can be utilized for specific situations. For example, if the task is to memorize a list of items, a participant may be taught a mnemonic strategy where to-be-remembered items are placed along a visual pathway, with vivid imagery solidifying the memory of the item along the path. Such a technique could allow the participant to recall important items for a presentation or examination. Both forms of cognitive remediation seem to benefit broad cognitive abilities of individuals with schizophrenia [16,19].

Although statistically there appear to be no differences in cognitive outcome between drill-and-practice and strategy-based cognitive remediation treatments in schizophrenia [16], other factors suggest that drill-and-practice procedures may represent the more sustainable long-term option. Drill-and-practice treatments tend to be less time consuming, are more likely to be computerized, and less likely to require direct contact with a therapist or mental health clinician [16]. Given these factors, it is likely that drill-and-practice cognitive remediation is more cost effective and less demanding on time both for participants patients and researchers/clinicians. However, cost remains a potential barrier for all parties. A cost effectiveness analysis indicated that a 40-session cognitive remediation program, including therapist time and all appropriate overhead and material costs, was over £630.00 per participant [29]. Given that these data were collected over a decade ago [30], expenses would now be substantially higher. Hence, despite drill-and-practice forms of cognitive remediation representing a more cost-effective option, cognitive remediation in any form can be a pricy endeavor. Thus, it is important to identify the most efficient forms of cognitive remediation for both trainee and training provider.

Further to the form of cognitive training that is most beneficial, an important consideration is the specific cognitive abilities to target. As noted, attention, working memory, and executive functioning were defined as key targets of cognitive remediation and are associated with the other factors important for occupational and social functioning: Social cognition and meta-cognition [16]. Targeting lower-level cognitive abilities such as attention and working memory, which are important for successful performance in higher-level processes such as executive functioning, social cognition, and meta-cognition, may be the more effective means of improving broader cognitive abilities and functional outcomes.

Returning to the definition of cognitive remediation for schizophrenia as provided at the 2010 Cognitive Remediation Experts Workshop [16], of note are the three key factors highlighted in that definition: That the training program is behavioral, that the training aims to improve cognitive processes, and that the training is durable and generalizable. The first factor, that training is behavioral, purposefully excludes cognitive remediation treatments based on medications (e.g., cholinesterase inhibitors) or brain stimulation (e.g., transcranial magnetic stimulation); therefore, these treatments are not covered in this review. While biological treatments have met with some success (see [31]), behaviorally based treatments are more common, more feasible to implement in the broad schizophrenia population, and are relatively convenient and side-effect free.

The remaining two facets of the definition of cognitive remediation ensure that the treatment indeed targets cognitive processes, although does not specify whether a particular cognitive process would be more beneficial to target. For example, enhancing attention may increase one’s ability to allow relevant information to enter and remain in working memory, which can impact executive functioning, social cognition, and metacognition. It is less likely, however, that enhancing one’s metacognitive abilities would transfer to enhanced attention. Perhaps the best strategy is to target a process with broad tendrils, such that enhancements result in bi-directional generalization to other abilities. For example, a training program that specifically targets working memory processes would, by its nature, train attentional processes, as well as generalize to more complex cognitive processes such as problem-solving. Hence, by targeting one domain, it may be possible to enhance all five aspects of cognition defined as targets of cognitive remediation.

One particular working memory training program has demonstrated promise in other populations as a program that enhances attention, working memory, and problem solving. This review will now turn to a discussion of findings of this working memory training program in healthy populations, with the intention of later recommending how this knowledge can be applied to schizophrenia.

## 3. Working Memory and Working Memory Training

Working memory is a central component of general cognition; however, the exact definition of working memory can vary based on the theoretical model followed. In an attempt to summarize commonalities among different models of working memory, Miyake and Shah [32] analyzed ten well-known models and determined that common to most models is the maintenance and processing of mental representations. Although terminology and exact representations vary among models (e.g., visual, auditory, speech, lexical, semantic, motor, mood, context, and/or tactile representations are described), most models recognize that working memory involves some form of temporary storage, as well as processing or manipulation of information [33,34,35,36,37,38].

It follows that working memory training aims to enhance an individual’s ability to temporarily store and process information. Both auditory and visual working memory are important for interpersonal day-to-day functioning of individuals with schizophrenia, including in social, occupational, educational, and community settings [15]. For example, well-functioning auditory working memory processes likely allow an individual to follow and respond to conversations and instructions, while visually working memory may be important for finding a job site or navigating an office environment, and manually manipulating objects to perform a task [15,39,40]. Given the known cognitive deficits in schizophrenia, and the potential implications of poor working memory in schizophrenia, it is surprising that more research has not focused specifically on working memory enhancement, whether specific to auditory or visual representations, or other representations (e.g., speech, motor, tactile, mood).

Although numerous working memory-training programs are available either in commercial (e.g., Lumosity, Nintendo BrainTrain, Jungle Memory, CogniFit) or clinical (e.g., Cogmed) settings, these programs are composed of a variety of training tasks targeting a number of cognitive domains. Hence, similar to cognitive remediation programs currently utilized and investigated in schizophrenia (see [16]), these programs do not target working memory processes specifically. Yet, targeted working memory training programs have demonstrated positive outcomes in enhancing various aspects of working memory, as well as attention and problem-solving, in a number of populations [41,42,43,44,45,46,47,48,49,50,51,52,53,54,55,56,57,58].

**Figure 1:**
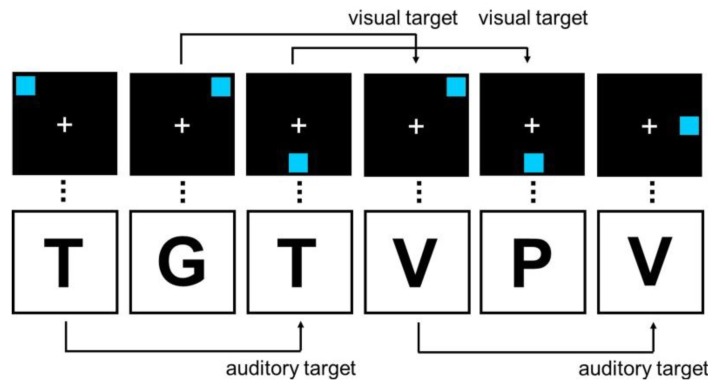
An example of a 2-back condition of a dual n-back task. Reproduced with permission from [41].

Dual n-back working memory training is a cognitively complex, adaptive, computerized training task that specifically targets working memory. The dual n-back training task, showing in Figure 1, requires trainees to simultaneously maintain and manipulate (*i.e.*, update) auditory and visual stimuli at increasing levels of difficulty. Specifically, trainees remember the position of an image on a grid, and an auditory stimulus such as a letter, word, or number. The trainee then responds based on whether the visual or auditory stimulus matches that presented n-back. The task can be accessed online as part of broader cognitive training programs (e.g., “Memory Lane” games available through subscription to www.lumosity.com [59]), or at no cost to users (e.g., [60]), thus, is an easily accessed and low cost alternative to more costly training programs.

## 4. Working Memory Training in Healthy Individuals

### 4.1. Cognitive Benefits

Multiple investigations in healthy populations support the use of dual n-back working memory training as a method to enhance not only working memory (referred to as near transfer), but also to generalize to cognitive domains beyond working memory (referred to as far transfer). Regarding near transfer, dual n-back working memory training has been credited for increased performance on tasks assessing recognition memory, immediate recall, and complex working memory abilities (e.g., reading span) in healthy adults relative to no-contact or active-control groups [41,42,43,44,45].

Regarding far transfer, the most commonly assessed outcome is fluid intelligence. Fluid intelligence is the ability to solve novel problems through reasoning, without reliance on crystalized (*i.e.*, previously acquired) knowledge [46]. Examples of such real-life, occupationally relevant problem solving includes figuring out how to take apart, fix, and reassemble a mechanical device, or coming up with a strategy to fix a computer problem. Fluid intelligence differs from working memory in that fluid intelligence invokes higher order cognitive processes such as comprehension and inferential reasoning, which allow for an understanding of associations among stimuli and the ability to solve problems related to those stimuli. However, fluid intelligence and working memory share capacity constraints, behavioral mechanisms, and common neural pathways in the frontal and parietal brain regions [47,48,49,50,51], which likely explains why several studies have demonstrated that working memory training impacts fluid intelligence. Specifically, Jaeggi and colleagues [52,53,54,55] have repeatedly demonstrated far transfer of dual n-back working memory training to measures of fluid intelligence in healthy adults, with these results replicated by other research teams [56,57,58].

Of note, not all dual n-back working memory training studies demonstrate transfer. In two studies of healthy young adults, dual n-back trainees failed to demonstrate gains in near or far transfer relative to an active visual search task control group or a no-contact control groups [61,62]. Similarly, young adults randomized to a dual n-back, visual 1-back, or no-contact control group did not show differential improvements after 8- or 20-days of training [63]. Finally, young adults who trained in a visual only dual n-back task did not improve in any cognitive measures relative to a Tetris playing control group [43]. Furthermore, several investigations have failed to identify dual n-back training related far transfer to fluid intelligence despite the presence of near transfer [41,42,44,45].

A number of factors may explain inconsistent results across dual n-back training studies. Of notable concern are the studies that utilized control tasks that targeted visual-spatial processing. In studies where an adaptive visual-spatial training task such as Tetris [43] or an adaptive visual search training task [61,62] was utilized, experimental and control tasks both employed adaptive visual-spatial training and thus the experimental and control training tasks may have been too similar to allow for differential effects to emerge. When a dual n-back task was compared to auditory only n-back, visual only n-back, visual short term memory training, and a no-training control group, far transfer to fluid intelligence was identified (near transfer was not measured) in the three training groups that employed a visual task, but not in the auditory or no-training control groups [56]. The authors suggested that the visuospatial component of training is a necessary ingredient of transfer to visual fluid intelligence [56].

Another potential reason for discrepancies across studies is the heterogeneity of study methodologies employed. For example, a variety of control groups have been used, including no-contact controls [41,42,54,55,57], sham controls utilizing a non-adaptive dual n-back task [63], and active controls training visual-spatial abilities [56,58,61,62], or general knowledge [45,52,53]. Additionally, baseline factors may have limited the ability to detect differential effects between experimental and control groups. For example, Thompson and colleagues [62] failed to identify enhancements in working memory or fluid intelligence after dual n-back training; however, both groups had a higher than average estimate of baseline intelligence (Wechsler Abbreviated Scale of Intelligence means of 121 in both groups, whereas average is 100). High baseline intelligence may interact with a ceiling effect and prevent the occurrence, or detection, of training related benefits. Similarly, a training group with higher baseline fluid intelligence scores compared to the control group is less likely to demonstrate training related change [41].

Finally, measures are inconsistent across studies. In these studies, the measures of working memory range from simple recall tasks, to complex working memory tasks requiring maintenance and simultaneous manipulation, as in reading span or operation span tasks. Additionally, visual fluid intelligence has been measured primarily with matrix-based tasks such as Raven’s or Bochumer Matrizen-Tests, though rarely with non-matrix problems. The overall heterogeneity of training protocols and study parameters likely accounts for the inconsistency across studies and, as a consequence, makes it difficult to clearly assess the value of dual n-back working memory training. In general, more methodologically rigorous investigations, which include identifying potential moderators or mediators of change, would both clarify and further knowledge about benefits of working memory training.

### 4.2. Neural Correlates

Further elucidation of the impact of working memory training can be garnered from brain structure data. The objectivity of structural data bypasses many limitations of behavioral-only studies, namely behavioral changes due to confounding variables such as inconsistent environmental factors, demand characteristics, placebo effects, or practice effects. Furthermore, identifying structural change can identify directions and regions of change in the absence of behavioural improvement.

In healthy adults, little is known about the extent and location of structural change associated with cognitive training, as most studies focus on specific skills development (e.g., juggling, mirror-reading) rather than more general cognitive ability. Studies investigating learning induced grey and white matter structural change have consistently demonstrated increases in grey and white matter volumes, which correspond to neural areas associated with the trained task, after a variety of different types of motor and/or cognitive training [64,65,66,67,68,69]. Specific to working memory training, grey matter volume changes have been noted after short periods (*i.e.*, five days) of training. In healthy young adults, training related changes were identified in the bilateral dorsolateral prefrontal cortex, right inferior parietal lobule, left paracentral lobule, and left superior temporal gyrus [70]. However, this is the only study to investigate structural change associated with working memory training, and the training period was both time limited (*i.e.*, five days) and not ecologically valid (*i.e.*, 4 h of training per day). In sum, there is a paucity of neuroanatomical studies related to working memory training, although existing evidence suggests that structural change is possible with repeated exposure to a cognitive task, such as the dual n-back.

Given the lack of neuroanatomical investigations specific to working memory training, hypothesized changes can be inferred through knowledge of neural correlates of working memory. Human lesion studies identified the orbitofrontal cortex and dorsolateral prefrontal cortex as key in working memory [71,72]. Barbey and colleagues assessed individuals with orbitofrontal cortex lesions, prefrontal cortex lesions, or no lesions on memory maintenance, memory manipulation, and fluid intelligence tasks. Orbitofrontal lesions were not associated with deficits on any measures of memory maintenance (e.g., simple span tasks) or manipulation (e.g., rearranging the order of letters or numbers being maintained in memory), or reasoning; however, deficits were present in measures that required the coordination of maintenance, manipulation, and monitoring/updating processes [71]. Similarly, when working memory and fluid intelligence was assessed in individuals with either a dorsolateral prefrontal cortex lesion, a non-dorsolateral prefrontal cortex lesion, or no brain lesion, participants performed equally on simple memory span tasks; however, those with left dorsolateral prefrontal cortex lesions had deficits in manipulation tasks. Furthermore, right dorsolateral prefrontal cortex lesions were associated with impaired fluid reasoning [72]. Taken together, the authors concluded that the orbitofrontal cortex is key for the executive control aspect of working memory, while the dorsolateral prefrontal cortex is important for working memory process and broader reasoning abilities [71,72]. Specifically, the left dorsolateral prefrontal cortex is associated with working memory and the right dorsolateral prefrontal cortex with reasoning [71,72]. Hence, we would expect that if working memory or fluid intelligence enhancements were to occur after cognitive training, such enhancements could be measured primarily in the orbital frontal cortex and dorsolateral prefrontal cortex. However, in order for changes in these areas to be expected after cognitive training, the training task must also be associated with these domains.

To date, there are no functional neuroimaging (fMRI) studies where dual n-back working memory training was the exclusive training paradigm. However, two reviews of fMRI studies which utilized other forms of working memory training either in a single session or across multiple training sessions (*i.e.*, 10–20 h training across several weeks) described changed activity in parietal, occipital, and dorsolateral areas after training [73,74]. For example, in a visuospatial n-back training task where participants trained for 28 consecutive days, increased parietal and dorsolateral prefrontal activations were identified at day 14, which decreased relative to baseline by day 28 [75]. Similarly, when healthy adults trained on a letter updating task for a total of 11 h across 5 weeks, increased caudate activity and decreased dorsolateral prefrontal and parietal activity was reported relative to untrained controls [76]. Of note is the multiple ways fMRI data related to training can be interpreted [74]. First, increased activity in a particular region may be due to increased cortical representation or more robust neural responding as more neural resources are allocated to that area. Conversely, increased efficiency of neural processes related to a task may result in decreased fMRI activity. Finally, activity may both increase and decrease across different neural regions associated with the training task (*i.e.*, activation redistribution) or new neural areas may be recruited and thus activate in response to a particular task (*i.e.*, activation reorganization) [74]. Hence, while training studies indicate alterations in expected areas due to training, it remains unclear what exactly these alterations mean.

## 5. Working Memory Training in Schizophrenia

### 5.1. Cognitive Benefits

Few studies focus specifically on working memory training in schizophrenia, and those that do emphasize auditory working memory. In a recent pilot study, in-patients with chronic schizophrenia underwent 4-weeks of computerized working memory training composed of visual-spatial working memory tasks and verbal working memory tasks. Relative to the non-trained controls, and despite the small sample size (*n* = 15 trainees), a statistically significant and large effect emerged for improved verbal working memory (*d* = 1.04) after training [77]. Furthermore, Fisher and colleagues’ [78] auditory training program, which included bottom-up processing training (e.g., distinction of basic auditory information such as sound frequencies and individual speech sounds such as phonemes and syllables) and practice with increasingly challenging verbal learning and working memory tasks (e.g., carrying out verbal instructions and remembering details from a conversation), was associated with increased verbal learning and memory and overall cognition after 10 weeks (50 h) of training [78]. Compared to more conventional forms of cognitive remediation (*i.e.*, as described by McGurk and colleagues [19]), effect sizes of targeted auditory working memory training were notably greater for global cognition (*i.e.*, *d* = 0.86* vs.* *d* = 0.41) and verbal tasks (*i.e.*, *d*’s = 0.58−0.89* vs.* *d*’s = 0.39−0.52) [78]. Importantly, gains made related to working memory training persisted six months after the intervention concluded [79]. Hence, preliminary evidence is emerging that auditory working memory training can positively impact auditory working memory, as well as more general cognitive tasks.

### 5.2. Neural Benefits

In schizophrenia patients, there are currently no dual n-back training studies. However, during n-back task performance, schizophrenia populations demonstrate changes in neural activity in a number of areas relative to healthy controls, suggesting these regions could be positively affected by working memory training. Specifically, hypoactivation is documented in the bilateral dorsolateral prefrontal cortex, rostral prefrontal cortex, and right ventrolateral/insular cortex, and hyperactivation in the right dorsomedial prefrontal cortex, left frontal pole, and the anterior cingulate [80].

Despite a paucity of studies, developing evidence suggests that working memory training has beneficial neural impacts for schizophrenia patients. Relative to controls, schizophrenia patients who trained with audio and visual processing exercises demonstrated increased medial prefrontal cortex activity after 80 h of training, with such activity associated with sustained improvements in psychosocial functioning [81,82] and working memory performance [82]. However, this training program included facial emotion recognition and theory of mind exercises; thus, it is difficult to determine which form of training was associated with neural benefit. Similarly, patients who utilized 6–8 weeks (17 h) of attention and working memory drill-and-practice training performed better on cognitive measures after training relative to healthy and placebo-trained patient controls, and displayed increased activity in neural networks associated with attention and working memory, specifically, the dorsolateral prefrontal cortex, anterior cingulate, and frontopolar cortex [83]. Although n-back training was included in the training protocols, the benefits of n-back training specifically were not isolated. Hence, while these studies suggest benefit to the neural networks of schizophrenia patients after working memory training, training programs remain broad, making it difficult to clearly determine what aspect of training is providing benefit. Furthermore, these studies were generally preliminary, thus, additional investigations with larger samples will better address the potential efficacy and effectiveness of working memory training on networks associated with attention and working memory in schizophrenia.

Wexler and colleagues [84] trained auditory working memory in schizophrenia patients using a verbal serial position memory task where a list of words was presented, then after a delay one of the words was provided and the trainee indicated in what position that word was in the list. As the participant achieved competence at the task (*i.e.*, 90% accuracy after three consecutive training days) the list length increased, thereby increasing difficulty of the task. Relative to before training, three participants improved on measures of verbal working memory after 10 weeks of training (20–30 h), with improvements corresponding to increased activation in the left inferior frontal cortex [84]. However, five participants failed to demonstrate notable improvement; the authors did not speculate as to why some patients benefited while others did not.

Although preliminary, the findings related to the behavioral and neural benefits of working memory training to verbal working memory are noteworthy given the importance of verbal working memory to social and work-related interpersonal interactions. For example, abilities such as remembering a newly introduced person’s name while having a conversation with that person, following multi-part instructions (e.g., “please page Dr. Smith then tell Sally to call Dr. Marks”), or responding to a two-part question (e.g., “where did you get the pipes from, how much were they, and what type of putty did you use to fix the leak?”) rely on auditory working memory processes. In an educational or employment setting, verbal working memory would be used while taking notes, or mentally forming and preparing to ask a question while still listening to the content of a presentation. Hence, enhanced verbal working memory can have notable implications on day-to-day functioning for individuals with schizophrenia navigating the complexities of social and occupational life. Using this knowledge, we suggest that investigators interested in better understanding the potential benefits of cognitive remediation in schizophrenia focus on working memory training as an important target for improvement.

## 6. Recommendations for Further Investigation

Given that the benefits of targeted working memory training have been relatively well established in healthy populations, it is a logical next step to extend research into working memory training to more vulnerable populations where a greater impact from training may be garnered. Although not directly investigated, it is possible that a population with known cognitive deficit is likely to improve more from working memory training relative to a healthy population with few or no cognitive complaints. Furthermore, from a clinical standpoint, improving the working memory abilities of individuals with schizophrenia clearly has wide-spread potential benefits in terms of occupational and social functioning. Hence, our first recommendation is to extend working memory training experiments to schizophrenia populations. To date, there are few studies reporting the effects of targeted working memory training in schizophrenia, although in all cases, effects of training on neurocognitive test performance were positive [77,78,79,82,83,84].

Despite positive performance on general neurocognitive training, assessment of the benefits of working memory training to real-life functioning has not been well considered for schizophrenia patients. Broader cognitive remediation studies have suggested small effects of training on positive and negative symptoms (*d* = 0.28) and general psychosocial functioning (*d* = 0.36) [19]. Numerous investigations of other forms of cognitive training, specifically processing speed training in non-psychotic older adults with developmentally appropriate slowed processing speed, identified benefits to driving ability and activities of daily living such as quickly finding items, looking up information, or determining correct change for cash transactions [85,86]. Working memory training itself has been associated with benefits such as enhanced school performance, specifically, improved reading comprehension [87]. Thus, computerized cognitive training programs appear to benefit a variety of individuals in daily activities not specifically trained by the program. However, at this point, we may only speculate upon how working memory training might improve activities of daily living in a schizophrenia population. Hence, including measures assessing day-to-day functional improvement attributable to working memory training is an important next step in determining the real world impact of training.

Furthermore, in order to strongly attribute improvement on neurocognitive tests or day-to-day functioning to the working memory training program, it will be important to compare working memory trainees to both an active control group and a no-contact control group. Such methodologically strong and well controlled investigations of the impact of working memory training in healthy populations are only now emerging; however, such factors are important in being able to identify the true extent to which working memory training benefits cognitive ability.

Little is known about the long-term benefits of training, as few studies include extensive follow-up periods. In a dual n-back training study in healthy children, near transfer to working memory improvement and far transfer to fluid intelligence improvement was maintained three months after training concluded [53]. In another study, healthy children with poorly developed working memory completed up to 25 days of a variety of drill-and-practice working memory tasks designed to target auditory or visual working memory (though never both simultaneously as in the dual n-back), trainees demonstrated sustained improvement in verbal and visual working memory, as well as mathematical reasoning six months after training concluded [88]. Of note, however, is that effect sizes appeared to decrease after 6-months relative to immediately post-training, suggesting that for training to be sustained in the longer term, continued practice with the tasks may be necessary. In schizophrenia patients, positive lasting impacts of training have been reported. Specifically, based on 11 studies where follow-up data were reported (follow-up timelines varied among studies and ranged from 3 to 24 months), effects of cognitive remediation on global cognition were maintained (*d* = 0.43) [16]. However, these 11 studies included a heterogeneous array of training programs, thus, identifying the long-term effect of any one form of training is challenging. In terms of working memory training specifically, only one study included a follow-up period for schizophrenia trainees. Fisher and colleagues (2010) reported sustained benefit to attention and verbal learning and memory six months after the completion of 50 h of auditory working memory training [79]. Among those who trained 100 h, improvements in processing speed and global cognition were maintained at a 6-month follow-up [79]. In a particularly vulnerable population such as individuals with schizophrenia, it will be important to elucidate both the dose and duration of training necessary. Specifically, it is prudent to identify how many training h are necessary and within what time period, as well as to further investigate whether benefits of short-term training are maintained or whether training must continue indefinitely. Alternatively, it is possible that periodic booster sessions would benefit the maintenance of training related gains. These are all viable areas of further investigation as such factors are presently not well understood.

In addition to dose and duration, patient adherence to training must be considered. When reported, adherence is high and attrition rates low (e.g., 3%–9% of participants removed or voluntarily withdrew) in laboratory based dual n-back training studies of healthy adults that offered completion incentives to each participant [53,55]. However, in a study where participants accessed training at a location of their choosing (e.g., home, office, or library) and were not offered individual incentives, attrition was substantially higher (28%) [89]. There are presently no dual n-back training studies specific to schizophrenia; adherence and attrition may present as special concerns within a schizophrenia population and are, thus, subject to investigation. Two potential issues with the dual n-back task may impact training compliance. First, the task is often repetitive (e.g., presentation of blocks and letters), hence, could become boring. Interest may be better maintained by varying the task stimuli while maintaining its dual n-back nature. For example, Lumosity’s [59] adaptation of the dual n-back task, Memory Lane, presents windows lighting up in the exterior of a building, while Rudebeck and colleagues’ [57] adaptation presents stimuli on walls in a three-dimensional room. Second, given the adaptive nature of dual n-back training, it may be important to consider whether immediately increasing task difficulty after good performance discourages participants, prompting low compliance and/or drop-out [89]. Allowing a participant to temporarily continue being successful at a newly achieved difficulty level may resolve this issue. However, this idea is speculative and has yet to be investigated.

Additionally, the mechanisms by which working memory training results in improvement remain unclear [28,90]. What components of working memory are impacted by training (e.g., attention, updating, manipulation)? In healthy adults, the effects of visual only n-back training match that of dual (auditory and visual) n-back training [54]. Would this finding extend to schizophrenia populations, or would individuals with schizophrenia benefit more or less from the more complex dual n-back form of training, relative to auditory or visual only n-back? Is it truly the training that benefits participants, or is it sustained attention to a challenging task? Do participants benefit from coming into a clinic for the training, or is training as effective if conducted at a time and location of the patient’s choosing? Are multiple training games needed to maintain interest, even if those games do not actually benefit the cognitive abilities of trainees? To what extent do individual differences moderate or mediate training related change? Such individual differences might include mood, psychotic symptoms, illness duration, medication profile, sleep, physical activity, baseline intelligence, baseline psychosocial functioning, education, or employment history. Clearly, both in the existing literature of working memory training in healthy populations and in the newly emerging area of working memory training for psychosis, numerous questions remain ripe for investigation.

## 7. Limitations

One purpose of this review was to summarize the published literature on working memory training in schizophrenia. While we identified six reports indicating beneficial impacts of working memory training on neurocognitive performance, it is possible that investigations have been conducted but not published due to null findings. An analysis of publication bias is beyond the scope of this review; however, the potential for bias is important to consider when drawing conclusions regarding the benefits of working memory training in schizophrenia.

Furthermore, in reporting neural effects of working memory training (e.g., structural and/or functional changes) it is important to acknowledge that pathological factors in schizophrenia (e.g., cerebral atrophy, enlarged ventricles) have not been clearly implicated in impaired cognitive performance [91]. Hence, one can neither assume that cerebral atrophy causes neurocognitive deficit, nor that working memory training alters the neural architecture specific to schizophrenia.

## 8. Conclusions

In sum, despite the numerous reviews and meta-analyses concluding positive impacts of general forms of cognitive remediation on individuals with schizophrenia, little is known about how more specific forms, such as working memory training, may be beneficial. Current programs are heterogeneous and resource heavy, potentially limiting accessibility. A more targeted and easily accessible form of computerized working memory training, adaptive n-back working memory training, has demonstrated positive behavioral and neural impacts in healthy populations. Such findings are beginning to emerge for individuals with schizophrenia, with potential benefits not only to aspects of cognition (e.g., attention, working memory, processing speed), but to more generalized functioning such as social and occupational performance. However, several questions remain regarding the details of training (e.g., dosage, duration, type), how training exerts an impact (e.g., improved attention, improved working memory), long term benefits of training, and how training must be adapted for the special needs of an individual with schizophrenia (e.g., impact of medication, symptoms, illness duration, and other individual differences).

There is ample room for well-controlled, methodologically rigorous investigations and attempts to identify moderators or mediators of training related benefit. Such work is necessary in order to extend working memory training to a population experiencing severe and persistent mental illness. Despite the likely benefits working memory training may produce in schizophrenia populations, empirical validation of such treatment is necessary before responsible recommendations can be made regarding working memory training in clinical and therapeutic settings. Furthermore, it will be important to identify a form of working memory training that is accessible, sustainable, and financially palatable for both affected individuals and service providers within public or private health systems.
